# Bis(μ_2_-2-amino-5-nitro­benzoato)bis­(2-amino-5-nitro­benzoato)octa­butyldi-μ_3_-oxido-tetra­tin(IV)

**DOI:** 10.1107/S1600536811028212

**Published:** 2011-07-23

**Authors:** Yip-Foo Win, Chen-Shang Choong, Siang-Guan Teoh, Chin Sing Yeap, Hoong-Kun Fun

**Affiliations:** a Department of Chemical Science, Faculty of Science, Universiti Tunku Abdul Rahman, Perak Campus, Jalan Universiti, Bandar Barat, 31900 Kampar, Perak, Malaysia; b School of Chemical Sciences, Universiti Sains Malaysia, 11800 USM, Penang, Malaysia; c X-ray Crystallography Unit, School of Physics, Universiti Sains Malaysia, 11800 USM, Penang, Malaysia

## Abstract

In the title complex, [Sn_4_(C_4_H_9_)_8_(C_7_H_5_N_2_O_4_)_4_O_2_], all four Sn^IV^ atoms are five-coordinated with distorted trigonal–bipyramidal SnC_2_O_3_ geometries. Two Sn^IV^ atoms are coordin­ated by two butyl groups, one benzoate O atom and two bridging O atoms, whereas the other two Sn^IV^ atoms are coordinated by two butyl groups, two benzoate O atoms and a bridging O atom. All the butyl groups are equatorial with respect to the SnO_3_ trigonal plane. In the crystal, mol­ecules are linked into a two-dimensional layer parallel to the *ab* plane by inter­molecular N—H⋯O and C—H⋯O hydrogen bonds and further stabilized by a π–π inter­action [centroid–centroid distance = 3.6489 (11) Å]. Intra­molecular N—H⋯O and C—H⋯O hydrogen bonds stabilize the mol­ecular structure. Two of the butyl groups are each disordered over two sets of sites with site-occupancy ratios of 0.510 (4):0.490 (4) and 0.860 (5):0.140 (5).

## Related literature

For general background to the title complex, see: Win *et al.* (2006 ▶); Win, Teoh *et al.* (2011 ▶). For closely related structures, see: Win *et al.* (2008 ▶); Win, Choong, Ha *et al.* (2010 ▶); Win, Choong *et al.* (2011 ▶); Win, Choong, Teoh *et al.* (2010 ▶). For the stability of the temperature controller used in the data collection, see: Cosier & Glazer (1986 ▶).
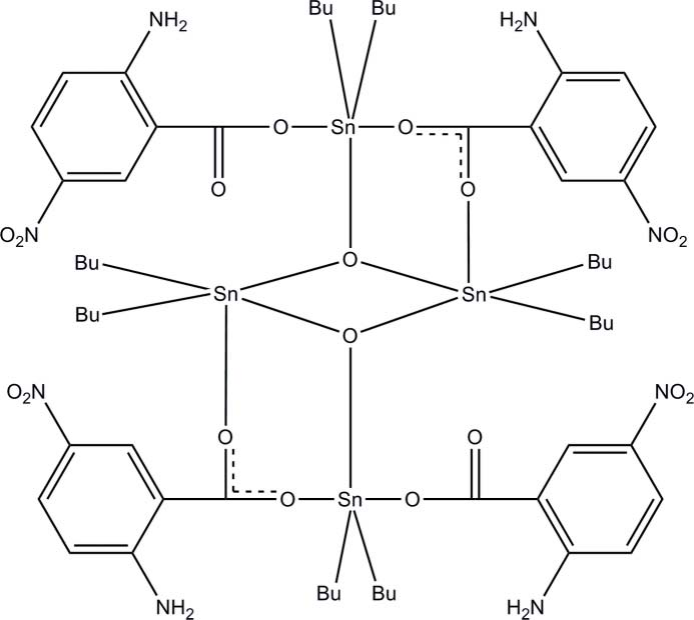


## Experimental

### 

#### Crystal data

[Sn_4_(C_4_H_9_)_8_(C_7_H_5_N_2_O_4_)_4_O_2_]
*M* *_r_* = 1688.18
Triclinic, 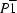

*a* = 14.3292 (2) Å
*b* = 15.3691 (2) Å
*c* = 18.2096 (2) Å
α = 80.289 (1)°
β = 74.982 (1)°
γ = 65.631 (1)°
*V* = 3519.49 (8) Å^3^
*Z* = 2
Mo *K*α radiation
μ = 1.47 mm^−1^
*T* = 100 K
0.45 × 0.19 × 0.07 mm

#### Data collection

Bruker SMART APEXII CCD area-detector diffractometer
Absorption correction: multi-scan (*SADABS*; Bruker, 2009 ▶) *T*_min_ = 0.554, *T*_max_ = 0.899
110407 measured reflections
29672 independent reflections
23074 reflections with *I* > 2σ(*I*)
*R*_int_ = 0.030

#### Refinement

*R*[*F* ^2^ > 2σ(*F* ^2^)] = 0.032
*wR*(*F* ^2^) = 0.073
*S* = 1.04
29672 reflections
906 parameters
H atoms treated by a mixture of independent and constrained refinement
Δρ_max_ = 2.67 e Å^−3^
Δρ_min_ = −1.48 e Å^−3^

### 

Data collection: *APEX2* (Bruker, 2009 ▶); cell refinement: *SAINT* (Bruker, 2009 ▶); data reduction: *SAINT*; program(s) used to solve structure: *SHELXTL* (Sheldrick, 2008 ▶); program(s) used to refine structure: *SHELXTL*; molecular graphics: *SHELXTL*; software used to prepare material for publication: *SHELXTL* and *PLATON* (Spek, 2009 ▶).

## Supplementary Material

Crystal structure: contains datablock(s) global, I. DOI: 10.1107/S1600536811028212/is2746sup1.cif

Structure factors: contains datablock(s) I. DOI: 10.1107/S1600536811028212/is2746Isup2.hkl

Additional supplementary materials:  crystallographic information; 3D view; checkCIF report

## Figures and Tables

**Table 1:** Hydrogen-bond geometry (Å, °)

*D*—H⋯*A*	*D*—H	H⋯*A*	*D*⋯*A*	*D*—H⋯*A*
N2—H1*N*2⋯O9^i^	0.86 (3)	2.32 (3)	3.122 (3)	157 (3)
N2—H2*N*2⋯O3	0.84 (3)	2.02 (3)	2.684 (3)	135 (2)
N4—H1*N*4⋯O6^ii^	0.84 (3)	2.20 (3)	3.002 (2)	160 (3)
N4—H2*N*4⋯O8	0.87 (3)	2.02 (3)	2.675 (2)	131 (3)
N6—H1*N*6⋯O12	0.82 (3)	2.07 (3)	2.688 (3)	132 (2)
N6—H2*N*6⋯O17^iii^	0.83 (3)	2.36 (3)	3.152 (3)	158 (2)
N6—H2*N*6⋯O18^iii^	0.83 (3)	2.49 (2)	3.229 (3)	149 (2)
N8—H1*N*8⋯O16	0.86 (3)	2.03 (3)	2.674 (2)	131 (3)
N8—H2*N*8⋯O13^iv^	0.82 (3)	2.22 (3)	2.998 (2)	160 (3)
C6—H6*A*⋯O9^i^	0.95	2.48	3.261 (2)	139
C13—H13*A*⋯O6^ii^	0.95	2.57	3.350 (2)	140
C20—H20*A*⋯O17^iii^	0.95	2.54	3.338 (3)	142
C43—H43*B*⋯O13^v^	0.99	2.58	3.544 (3)	164
C50—H50*B*⋯O8	0.99	2.56	3.304 (3)	131
C54—H54*A*⋯O16	0.99	2.51	3.210 (3)	128
C58—H58*A*⋯O16	0.99	2.58	3.221 (2)	123

Symmetry codes: (i) 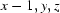; (ii) 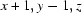; (iii) 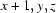; (iv) 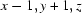; (v) 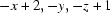.

**Fig. 1.**
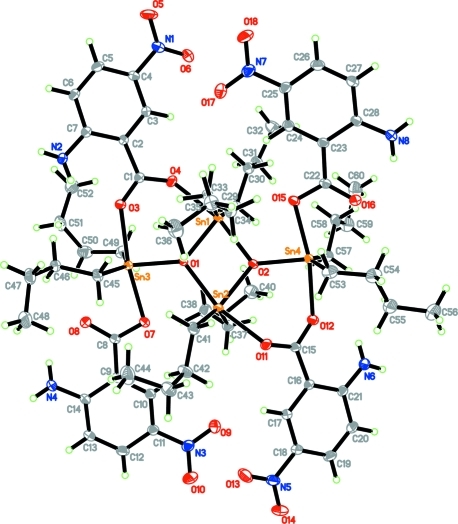
The molecular structure of the title compound, with 50% probability ellipsoids for non-H atoms. Minor disorder components were omitted for clarity.

**Fig. 2.**
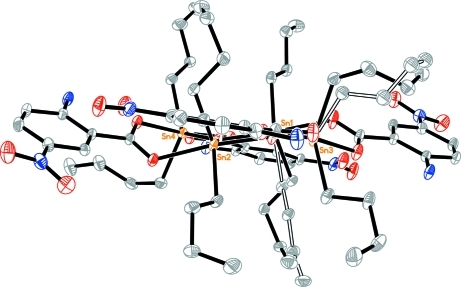
The molecular structure of the title compound without hydrogen atoms, showing all disorder components. Hydrogen atoms are omitted for clarity.

**Fig. 3.**
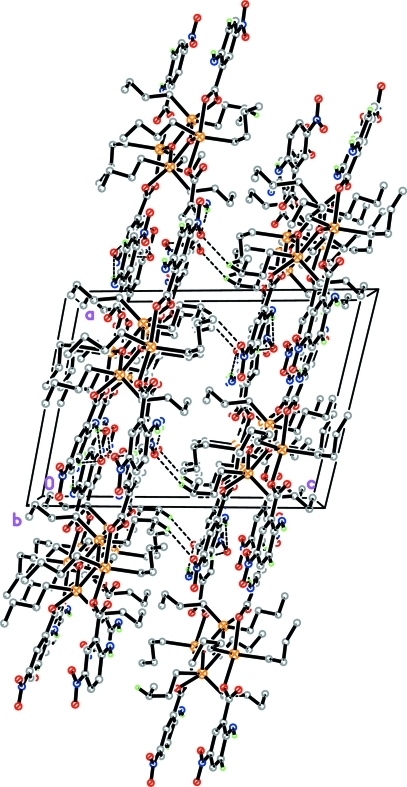
The crystal structure of the title compound, showing a two-dimensional plane parallel to the (0 0 1) plane. Hydrogen atoms not involved in hydrogen bonds (dashed lines) and minor disorder components are omitted for clarity.

## supplementary crystallographic information

### Comment

In general, diorganotin(IV) carboxylate complexes can be obtained in monomeric or organodistannoxane dimer forms when the reaction of diorganotin(IV) with carboxylic acid is carried out in 1:2 or 1:1 ratio respectively (Win *et al.*, 2006; Win, Teoh *et al.*, 2011). The core geometry of the organodistannoxane dimer complexes consists of a centrosymmetric planar Sn_2_O_2_ group and all the four tin(IV) atoms (*exo*- and endocyclic) are five coordinated and exist in distorted trigonal bipyramidal geometry (Win *et al.*, 2008; Win, Choong, Ha *et al.*, 2010; Win, Choong *et al.*, 2011; Win, Choong, Teoh *et al.*, 2010). In this study, the structure of the title complex (I) is in dimeric form whereas bis(2-amino-5-nitrobenzoato-*κ*^2^*O*,*O*')dibutyltin(IV) is in monomeric structure.

Similar to previous structures (Win *et al.*, 2008; Win, Choong, Ha *et al.*, 2010; Win, Choong *et al.*, 2011; Win, Choong, Teoh *et al.*, 2010), all Sn atoms are five-coordinated in distorted trigonal-bipiramidal geometries (Fig. 1). The Sn1 and Sn2 atoms are coordinated by two butyl groups in equatorial positions, an O atom of the bridging benzoate anion and two oxido-bridged O atoms whereas the Sn3 and Sn4 atoms are coordinated by two butyl groups in equatorial positions, an O atom of the monodentate benzoate anion, an O atom of the bridging benzoate anion and one oxo-bridged O atom. In the crystal structure, the molecules are linked into two-dimensional planes parallel to (0 0 1) plane (Fig. 3) by intermolecular N—H···O and C—H···O hydrogen bonds (Table 1). The crystal structure is further stabilized by the *Cg*1···*Cg*2 interaction of 3.6489 (11) Å, -1 + *x*, *y*, *z* (*Cg*1 and *Cg*2 are centroids of benzene ring C2–C7 and C16–C21). Intramolecular N—H···O and C—H···O hydrogen bonds (Table 1) stabilize the molecular structure.

### Experimental

The title complex was prepared from a 1:1 molar mixture of dibutyltin(IV) oxide (0.50 g, 2 mmole) and 2-amino-5-nitrobenzoic acid (0.36 g, 2 mmole) in ethanol (50 ml). The resulting mixture was heated under reflux for two hours. A clear yellow transparent solution was isolated by filtration and kept in a bottle. After four days, yellow solids (0.61 g, 73.0% yield) were collected. Melting point: 240.7–241.5 °C. Analysis for C_60_H_92_N_8_O_18_Sn_4_: C, 42.74; H, 5.79; N, 6.57; Sn, 27.98%. Calculated for C_60_H_92_N_8_O_18_Sn_4_: C, 42.66; H, 5.49; N, 6.64; Sn, 28.12%. FTIR as KBr disc (cm^-1^): *v*(NH_2_) 3457, 3344, 3314; *v*(C—H) aromatic 3059, *v*(C—H) saturated 2956, 2926, 2870; *v*(COO)_as_ 1622, *v*(COO)_s_ 1310, *v*(NO_2_) 1537, *v*(Sn—O—Sn) 630, *v*(Sn—C) 531, *v*(Sn—O) 391. ^1^H-NMR (p.p.m.) (*d*_6_-DMSO): δ: benzene protons 6.92 (d, 9.3 Hz, 4H); 8.12 (dd, 2.4 Hz, 9.2 Hz, 4H); 8.72 (s, 4H); butyl, CH_3_ 0.84 (t, 7.3 Hz, 12H); 0.90 (t, 7.3 Hz, 12H); CH_2_ 1.28–1.43 (m, 32H); CH_2_ 1.64–1.80 (m, 16H). ^13^C-NMR (p.p.m.) (*d*_6_-DMSO): δ: benzene carbons 112.27, 116.50, 128.49, 129.47, 135.55, 156.49; butyl 13.71, 13.91, 26.09, 26.77, 27.02, 27.29, 29.90; COO 172.11. ^119^Sn-NMR (p.p.m.) (*d*_6_-DMSO): δ: -173.87, -213.71.

### Refinement

All hydrogen atoms were positioned geomatrically (C—H = 0.95–0.99 Å) and refined using a riding model, with *U*_iso_(H) = 1.2 or 1.5*U*_eq_(C). A rotating-group model was applied for methyl groups. Two of the butyl groups are disordered over two positions with refined site-occupancy ratios of 0.510 (4):0.490 (4) and 0.860 (5):0.140 (5) (Fig. 2). The same *U*_ij_ parameters was used for atom pair C50X/C52X. The C49X, C50X, C51X and C52X atoms were refined isotropically. The maximum and minimum residual electron density peaks of 2.67 and -1.48 e Å^-3^ were located 0.66 and 0.53 Å, respectively, from atom Sn3 . Four reflections, (7 -5 22), (-2 16 18), (5 -15 14) and (0 0 2), were omitted.

### Crystal data

**Table:**

[Sn_4_(C_4_H_9_)_8_(C_7_H_5_N_2_O_4_)_4_O_2_]	*Z* = 2
*M**_r_* = 1688.18	*F*(000) = 1704
Triclinic, *P*1	*D*_x_ = 1.593 Mg m^−^^3^
Hall symbol: -P 1	Mo *K*α radiation, λ = 0.71073 Å
*a* = 14.3292 (2) Å	Cell parameters from 9105 reflections
*b* = 15.3691 (2) Å	θ = 2.3–34.5°
*c* = 18.2096 (2) Å	µ = 1.47 mm^−^^1^
α = 80.289 (1)°	*T* = 100 K
β = 74.982 (1)°	Block, yellow
γ = 65.631 (1)°	0.45 × 0.19 × 0.07 mm
*V* = 3519.49 (8) Å^3^	

### Data collection

**Table:**

Bruker SMART APEXII CCD area-detector diffractometer	29672 independent reflections
Radiation source: fine-focus sealed tube	23074 reflections with *I* > 2σ(*I*)
graphite	*R*_int_ = 0.030
φ and ω scans	θ_max_ = 34.7°, θ_min_ = 1.2°
Absorption correction: multi-scan (*SADABS*; Bruker, 2009)	*h* = −21→22
*T*_min_ = 0.554, *T*_max_ = 0.899	*k* = −24→24
110407 measured reflections	*l* = −29→29

### Refinement

**Table:**

Refinement on *F*^2^	Primary atom site location: structure-invariant direct methods
Least-squares matrix: full	Secondary atom site location: difference Fourier map
*R*[*F*^2^ > 2σ(*F*^2^)] = 0.032	Hydrogen site location: inferred from neighbouring sites
*wR*(*F*^2^) = 0.073	H atoms treated by a mixture of independent and constrained refinement
*S* = 1.04	*w* = 1/[σ^2^(*F*_o_^2^) + (0.0254*P*)^2^ + 2.328*P*] where *P* = (*F*_o_^2^ + 2*F*_c_^2^)/3
29672 reflections	(Δ/σ)_max_ = 0.003
906 parameters	Δρ_max_ = 2.67 e Å^−^^3^
0 restraints	Δρ_min_ = −1.48 e Å^−^^3^

### Special details

**Table:**

Experimental. The crystal was placed in the cold stream of an Oxford Cryosystems Cobra open-flow nitrogen cryostat (Cosier & Glazer, 1986) operating at 100.0 (1) K.
Geometry. All e.s.d.'s (except the e.s.d. in the dihedral angle between two l.s. planes) are estimated using the full covariance matrix. The cell e.s.d.'s are taken into account individually in the estimation of e.s.d.'s in distances, angles and torsion angles; correlations between e.s.d.'s in cell parameters are only used when they are defined by crystal symmetry. An approximate (isotropic) treatment of cell e.s.d.'s is used for estimating e.s.d.'s involving l.s. planes.
Refinement. Refinement of *F*^2^ against ALL reflections. The weighted *R*-factor *wR* and goodness of fit *S* are based on *F*^2^, conventional *R*-factors *R* are based on *F*, with *F* set to zero for negative *F*^2^. The threshold expression of *F*^2^ > σ(*F*^2^) is used only for calculating *R*-factors(gt) *etc*. and is not relevant to the choice of reflections for refinement. *R*-factors based on *F*^2^ are statistically about twice as large as those based on *F*, and *R*- factors based on ALL data will be even larger.

### Fractional atomic coordinates and isotropic or equivalent isotropic displacement parameters (Å^2^)

**Table:**

	*x*	*y*	*z*	*U*_iso_*/*U*_eq_	Occ. (<1)
Sn1	0.615883 (9)	0.312195 (8)	0.235446 (7)	0.01671 (3)	
Sn2	0.844716 (8)	0.170539 (8)	0.276260 (7)	0.01463 (3)	
Sn3	0.726881 (9)	0.058100 (8)	0.183349 (8)	0.01861 (3)	
Sn4	0.737168 (9)	0.429485 (8)	0.317647 (7)	0.01379 (2)	
O1	0.72397 (9)	0.17492 (8)	0.22430 (8)	0.0187 (2)	
O2	0.73553 (9)	0.30762 (8)	0.28821 (7)	0.0172 (2)	
O3	0.54677 (10)	0.12840 (9)	0.19435 (8)	0.0225 (3)	
O4	0.52363 (11)	0.28179 (10)	0.16622 (10)	0.0287 (3)	
O5	0.06846 (11)	0.48611 (11)	0.12433 (10)	0.0354 (4)	
O6	0.20536 (12)	0.51919 (10)	0.11020 (9)	0.0289 (3)	
O7	0.89082 (10)	0.00652 (9)	0.19418 (8)	0.0192 (2)	
O8	0.88365 (10)	−0.11355 (10)	0.14687 (8)	0.0242 (3)	
O9	1.21758 (12)	−0.00267 (10)	0.22356 (9)	0.0292 (3)	
O10	1.35710 (11)	−0.13356 (11)	0.21556 (9)	0.0318 (3)	
O11	0.92936 (10)	0.20281 (9)	0.35023 (7)	0.0187 (2)	
O12	0.91575 (10)	0.35208 (9)	0.31013 (8)	0.0190 (2)	
O13	1.24913 (12)	−0.04123 (10)	0.39659 (9)	0.0298 (3)	
O14	1.39161 (12)	−0.01430 (11)	0.37262 (10)	0.0353 (4)	
O15	0.57450 (9)	0.48247 (9)	0.30492 (7)	0.0176 (2)	
O16	0.58029 (10)	0.60183 (9)	0.35400 (7)	0.0195 (2)	
O17	0.24990 (12)	0.48413 (11)	0.27848 (10)	0.0312 (3)	
O18	0.10145 (12)	0.59988 (12)	0.31533 (11)	0.0367 (4)	
N1	0.16269 (12)	0.46123 (12)	0.12192 (10)	0.0225 (3)	
N2	0.39713 (13)	0.07371 (11)	0.18058 (10)	0.0211 (3)	
N3	1.26428 (12)	−0.08903 (12)	0.21179 (9)	0.0211 (3)	
N4	1.04704 (14)	−0.28197 (12)	0.14185 (11)	0.0248 (3)	
N5	1.29631 (13)	0.01373 (12)	0.37831 (10)	0.0235 (3)	
N6	1.07285 (13)	0.40233 (12)	0.31479 (10)	0.0212 (3)	
N7	0.19774 (13)	0.56302 (12)	0.30606 (10)	0.0254 (3)	
N8	0.40377 (14)	0.75500 (12)	0.39207 (10)	0.0229 (3)	
C1	0.49264 (14)	0.21414 (13)	0.17427 (11)	0.0194 (3)	
C2	0.38675 (13)	0.23784 (12)	0.15991 (10)	0.0155 (3)	
C3	0.32634 (13)	0.33358 (12)	0.14445 (10)	0.0166 (3)	
H3A	0.3545	0.3807	0.1409	0.020*	
C4	0.22574 (13)	0.36082 (12)	0.13420 (10)	0.0181 (3)	
C5	0.18189 (14)	0.29339 (14)	0.13872 (11)	0.0208 (3)	
H5A	0.1122	0.3131	0.1322	0.025*	
C6	0.24094 (14)	0.19865 (13)	0.15260 (11)	0.0197 (3)	
H6A	0.2121	0.1525	0.1542	0.024*	
C7	0.34398 (13)	0.16758 (12)	0.16469 (10)	0.0163 (3)	
C8	0.93483 (13)	−0.07913 (12)	0.17050 (11)	0.0181 (3)	
C9	1.04682 (13)	−0.13431 (12)	0.17266 (10)	0.0165 (3)	
C10	1.10336 (13)	−0.08855 (12)	0.19018 (10)	0.0164 (3)	
H10A	1.0698	−0.0229	0.2007	0.020*	
C11	1.20830 (13)	−0.13792 (13)	0.19243 (10)	0.0178 (3)	
C12	1.26059 (14)	−0.23398 (13)	0.17577 (11)	0.0214 (4)	
H12A	1.3328	−0.2668	0.1768	0.026*	
C13	1.20658 (15)	−0.28020 (13)	0.15796 (11)	0.0222 (4)	
H13A	1.2423	−0.3453	0.1461	0.027*	
C14	1.09805 (14)	−0.23306 (12)	0.15676 (10)	0.0185 (3)	
C15	0.96620 (13)	0.26731 (12)	0.33423 (10)	0.0156 (3)	
C16	1.07532 (13)	0.24094 (12)	0.34266 (10)	0.0153 (3)	
C17	1.13392 (13)	0.14435 (12)	0.35820 (10)	0.0164 (3)	
H17A	1.1025	0.0990	0.3653	0.020*	
C18	1.23695 (14)	0.11414 (13)	0.36337 (10)	0.0193 (3)	
C19	1.28521 (14)	0.17921 (14)	0.35424 (11)	0.0219 (4)	
H19A	1.3565	0.1573	0.3577	0.026*	
C20	1.22869 (14)	0.27455 (14)	0.34031 (11)	0.0202 (3)	
H20A	1.2607	0.3189	0.3359	0.024*	
C21	1.12296 (13)	0.30873 (13)	0.33225 (10)	0.0164 (3)	
C22	0.52883 (13)	0.56598 (12)	0.33300 (10)	0.0159 (3)	
C23	0.41350 (13)	0.61543 (12)	0.34005 (10)	0.0155 (3)	
C24	0.35931 (14)	0.57013 (12)	0.31860 (10)	0.0178 (3)	
H24A	0.3967	0.5097	0.2974	0.021*	
C25	0.25170 (14)	0.61226 (13)	0.32779 (11)	0.0192 (3)	
C26	0.19400 (14)	0.70151 (14)	0.35846 (11)	0.0225 (4)	
H26A	0.1198	0.7294	0.3651	0.027*	
C27	0.24589 (14)	0.74797 (13)	0.37865 (11)	0.0216 (4)	
H27A	0.2070	0.8089	0.3988	0.026*	
C28	0.35682 (13)	0.70719 (12)	0.37021 (10)	0.0173 (3)	
C29	0.64551 (14)	0.39952 (12)	0.13608 (11)	0.0191 (3)	
H29A	0.7014	0.4193	0.1404	0.023*	
H29B	0.6729	0.3602	0.0916	0.023*	
C30	0.55173 (14)	0.48939 (13)	0.12021 (11)	0.0201 (3)	
H30A	0.5282	0.5325	0.1618	0.024*	
H30B	0.4933	0.4711	0.1197	0.024*	
C31	0.57773 (16)	0.54309 (13)	0.04414 (11)	0.0228 (4)	
H31A	0.6420	0.5538	0.0419	0.027*	
H31B	0.5923	0.5030	0.0021	0.027*	
C32	0.48907 (18)	0.63928 (15)	0.03270 (12)	0.0308 (5)	
H32A	0.5051	0.6668	−0.0193	0.046*	
H32B	0.4818	0.6829	0.0693	0.046*	
H32C	0.4234	0.6299	0.0407	0.046*	
C33	0.5020 (6)	0.2954 (5)	0.3247 (3)	0.0172 (10)	0.510 (4)
H33A	0.4337	0.3458	0.3175	0.021*	0.510 (4)
H33B	0.4989	0.2329	0.3219	0.021*	0.510 (4)
C34	0.5145 (3)	0.2996 (3)	0.4045 (2)	0.0198 (8)	0.510 (4)
H34A	0.4913	0.3674	0.4151	0.024*	0.510 (4)
H34B	0.5895	0.2669	0.4068	0.024*	0.510 (4)
C35	0.4506 (3)	0.2521 (3)	0.4662 (2)	0.0222 (8)	0.510 (4)
H35A	0.4445	0.2726	0.5165	0.027*	0.510 (4)
H35B	0.3790	0.2753	0.4566	0.027*	0.510 (4)
C36	0.4977 (4)	0.1437 (3)	0.4690 (3)	0.0334 (11)	0.510 (4)
H36A	0.4505	0.1185	0.5066	0.050*	0.510 (4)
H36B	0.5657	0.1197	0.4837	0.050*	0.510 (4)
H36C	0.5074	0.1226	0.4187	0.050*	0.510 (4)
C33X	0.5016 (6)	0.3117 (5)	0.3451 (4)	0.0197 (11)	0.490 (4)
H33C	0.5049	0.3525	0.3802	0.024*	0.490 (4)
H33D	0.4297	0.3390	0.3354	0.024*	0.490 (4)
C34X	0.5250 (3)	0.2105 (3)	0.3824 (2)	0.0204 (8)	0.490 (4)
H34C	0.5976	0.1829	0.3905	0.025*	0.490 (4)
H34D	0.5204	0.1704	0.3473	0.025*	0.490 (4)
C35X	0.4502 (4)	0.2068 (3)	0.4586 (2)	0.0246 (9)	0.490 (4)
H35C	0.4602	0.2408	0.4956	0.030*	0.490 (4)
H35D	0.3770	0.2404	0.4517	0.030*	0.490 (4)
C36X	0.4675 (4)	0.1043 (3)	0.4908 (3)	0.0313 (10)	0.490 (4)
H36D	0.4142	0.1051	0.5373	0.047*	0.490 (4)
H36E	0.5373	0.0728	0.5027	0.047*	0.490 (4)
H36F	0.4620	0.0691	0.4530	0.047*	0.490 (4)
C37	0.96067 (14)	0.18908 (13)	0.18150 (10)	0.0191 (3)	
H37A	0.9601	0.2535	0.1824	0.023*	
H37B	1.0300	0.1419	0.1897	0.023*	
C38	0.95222 (16)	0.17938 (15)	0.10179 (11)	0.0244 (4)	
H38A	0.8778	0.2104	0.0978	0.029*	
H38B	0.9770	0.1107	0.0936	0.029*	
C39	1.01647 (16)	0.22486 (15)	0.03967 (11)	0.0261 (4)	
H39A	1.0870	0.2042	0.0507	0.031*	
H39B	1.0255	0.2008	−0.0099	0.031*	
C40	0.96720 (18)	0.33400 (15)	0.03267 (12)	0.0296 (4)	
H40A	1.0127	0.3583	−0.0075	0.044*	
H40B	0.9588	0.3586	0.0812	0.044*	
H40C	0.8985	0.3552	0.0198	0.044*	
C41	0.82967 (14)	0.06716 (13)	0.36786 (11)	0.0199 (3)	
H41A	0.7955	0.0998	0.4159	0.024*	
H41B	0.7838	0.0382	0.3588	0.024*	
C42	0.93581 (15)	−0.01281 (13)	0.37689 (11)	0.0240 (4)	
H42A	0.9678	−0.0476	0.3298	0.029*	
H42B	0.9830	0.0168	0.3826	0.029*	
C43	0.92831 (17)	−0.08493 (14)	0.44502 (11)	0.0259 (4)	
H43A	1.0000	−0.1274	0.4516	0.031*	
H43B	0.8913	−0.0497	0.4916	0.031*	
C44	0.8716 (2)	−0.14621 (16)	0.43712 (14)	0.0355 (5)	
H44A	0.8760	−0.1949	0.4799	0.053*	
H44B	0.9043	−0.1776	0.3891	0.053*	
H44C	0.7979	−0.1057	0.4373	0.053*	
C45	0.68765 (15)	−0.03894 (13)	0.27141 (12)	0.0231 (4)	
H45A	0.7491	−0.0768	0.2947	0.028*	
H45B	0.6299	−0.0020	0.3115	0.028*	
C46	0.65399 (16)	−0.10772 (13)	0.24266 (13)	0.0256 (4)	
H46A	0.5888	−0.0700	0.2237	0.031*	
H46B	0.7090	−0.1400	0.1992	0.031*	
C47	0.63512 (16)	−0.18382 (14)	0.30332 (13)	0.0287 (4)	
H47A	0.5859	−0.1518	0.3490	0.034*	
H47B	0.6013	−0.2171	0.2837	0.034*	
C48	0.7341 (2)	−0.25768 (16)	0.32661 (15)	0.0371 (5)	
H48A	0.7178	−0.3086	0.3605	0.056*	
H48B	0.7624	−0.2270	0.3533	0.056*	
H48C	0.7862	−0.2852	0.2811	0.056*	
C49	0.75241 (19)	0.08989 (19)	0.06048 (14)	0.0246 (5)	0.860 (5)
H49A	0.6955	0.1512	0.0495	0.030*	0.860 (5)
H49C	0.8190	0.0998	0.0434	0.030*	0.860 (5)
C50	0.75720 (19)	0.01522 (19)	0.01225 (14)	0.0287 (6)	0.860 (5)
H50A	0.7807	0.0332	−0.0421	0.034*	0.860 (5)
H50B	0.8109	−0.0473	0.0254	0.034*	0.860 (5)
C51	0.6545 (2)	0.00274 (17)	0.02148 (15)	0.0274 (6)	0.860 (5)
H51A	0.6681	−0.0519	−0.0073	0.033*	0.860 (5)
H51B	0.6298	−0.0135	0.0760	0.033*	0.860 (5)
C52	0.5679 (2)	0.0905 (2)	−0.00562 (17)	0.0287 (6)	0.860 (5)
H52A	0.5055	0.0764	0.0004	0.043*	0.860 (5)
H52B	0.5917	0.1077	−0.0595	0.043*	0.860 (5)
H52C	0.5507	0.1441	0.0247	0.043*	0.860 (5)
C49X	0.7335 (13)	0.0636 (12)	0.0793 (9)	0.023 (3)*	0.140 (5)
H49B	0.6881	0.1290	0.0633	0.028*	0.140 (5)
H49D	0.8063	0.0527	0.0520	0.028*	0.140 (5)
C50X	0.7001 (13)	−0.0089 (11)	0.0539 (9)	0.029 (3)*	0.140 (5)
H50C	0.7556	−0.0734	0.0588	0.035*	0.140 (5)
H50D	0.6357	−0.0093	0.0905	0.035*	0.140 (5)
C51X	0.6803 (13)	0.0057 (12)	−0.0225 (10)	0.030 (4)*	0.140 (5)
H51C	0.6740	−0.0521	−0.0342	0.035*	0.140 (5)
H51D	0.7410	0.0134	−0.0596	0.035*	0.140 (5)
C52X	0.5818 (17)	0.0924 (16)	−0.0327 (11)	0.029 (3)*	0.140 (5)
H52D	0.5658	0.0920	−0.0818	0.043*	0.140 (5)
H52E	0.5928	0.1509	−0.0316	0.043*	0.140 (5)
H52F	0.5231	0.0903	0.0086	0.043*	0.140 (5)
C53	0.71291 (16)	0.41534 (15)	0.43874 (11)	0.0243 (4)	
H53A	0.6413	0.4169	0.4599	0.029*	
H53B	0.7628	0.3516	0.4540	0.029*	
C54	0.72566 (16)	0.49149 (16)	0.47538 (11)	0.0273 (4)	
H54A	0.6736	0.5551	0.4623	0.033*	
H54B	0.7096	0.4794	0.5315	0.033*	
C55	0.83427 (17)	0.49452 (16)	0.45111 (13)	0.0299 (4)	
H55A	0.8477	0.5130	0.3958	0.036*	
H55B	0.8871	0.4295	0.4596	0.036*	
C56	0.8481 (2)	0.56420 (19)	0.49380 (14)	0.0392 (6)	
H56A	0.9191	0.5633	0.4753	0.059*	
H56B	0.8374	0.5451	0.5485	0.059*	
H56C	0.7968	0.6290	0.4851	0.059*	
C57	0.79555 (14)	0.50760 (13)	0.22150 (10)	0.0197 (3)	
H57A	0.8425	0.5299	0.2375	0.024*	
H57B	0.8397	0.4617	0.1821	0.024*	
C58	0.71941 (15)	0.59374 (12)	0.18323 (10)	0.0193 (3)	
H58A	0.6764	0.6423	0.2208	0.023*	
H58B	0.6716	0.5734	0.1663	0.023*	
C59	0.77550 (18)	0.63886 (16)	0.11475 (12)	0.0310 (5)	
H59A	0.8171	0.5906	0.0767	0.037*	
H59B	0.8248	0.6573	0.1316	0.037*	
C60	0.7005 (2)	0.72705 (16)	0.07678 (13)	0.0352 (5)	
H60A	0.7410	0.7555	0.0357	0.053*	
H60B	0.6567	0.7740	0.1147	0.053*	
H60C	0.6559	0.7082	0.0556	0.053*	
H1N2	0.363 (2)	0.0372 (19)	0.1916 (15)	0.038 (7)*	
H2N2	0.456 (2)	0.0581 (17)	0.1905 (13)	0.026 (6)*	
H1N4	1.078 (2)	−0.340 (2)	0.1326 (15)	0.040 (8)*	
H2N4	0.980 (2)	−0.2534 (19)	0.1430 (15)	0.038 (7)*	
H1N6	1.013 (2)	0.4218 (19)	0.3087 (15)	0.036 (7)*	
H2N6	1.1052 (18)	0.4383 (17)	0.3072 (13)	0.022 (6)*	
H1N8	0.471 (2)	0.733 (2)	0.3835 (17)	0.052 (9)*	
H2N8	0.3716 (19)	0.8117 (18)	0.3995 (13)	0.026 (6)*	

### Atomic displacement parameters (Å^2^)

**Table:**

	*U*^11^	*U*^22^	*U*^33^	*U*^12^	*U*^13^	*U*^23^
Sn1	0.01106 (5)	0.00921 (5)	0.03082 (6)	−0.00378 (4)	−0.00783 (4)	0.00106 (4)
Sn2	0.01039 (5)	0.00965 (5)	0.02354 (6)	−0.00362 (4)	−0.00501 (4)	0.00118 (4)
Sn3	0.01384 (5)	0.01048 (5)	0.03358 (7)	−0.00422 (4)	−0.00888 (5)	−0.00213 (4)
Sn4	0.01225 (5)	0.01044 (5)	0.01863 (5)	−0.00414 (4)	−0.00414 (4)	−0.00019 (4)
O1	0.0132 (5)	0.0096 (5)	0.0352 (7)	−0.0031 (4)	−0.0103 (5)	−0.0016 (5)
O2	0.0122 (5)	0.0095 (5)	0.0300 (7)	−0.0029 (4)	−0.0071 (5)	−0.0009 (5)
O3	0.0150 (6)	0.0129 (6)	0.0412 (8)	−0.0047 (5)	−0.0108 (5)	−0.0001 (5)
O4	0.0239 (7)	0.0152 (6)	0.0561 (10)	−0.0105 (5)	−0.0225 (7)	0.0039 (6)
O5	0.0163 (7)	0.0268 (8)	0.0557 (10)	−0.0004 (6)	−0.0130 (7)	0.0038 (7)
O6	0.0260 (7)	0.0141 (6)	0.0465 (9)	−0.0047 (5)	−0.0120 (7)	−0.0034 (6)
O7	0.0150 (6)	0.0118 (5)	0.0305 (7)	−0.0029 (5)	−0.0072 (5)	−0.0028 (5)
O8	0.0184 (6)	0.0190 (6)	0.0383 (8)	−0.0052 (5)	−0.0114 (6)	−0.0070 (6)
O9	0.0282 (7)	0.0197 (7)	0.0452 (9)	−0.0120 (6)	−0.0137 (7)	0.0000 (6)
O10	0.0183 (7)	0.0346 (8)	0.0457 (9)	−0.0115 (6)	−0.0109 (6)	−0.0014 (7)
O11	0.0170 (6)	0.0166 (6)	0.0257 (6)	−0.0091 (5)	−0.0081 (5)	0.0027 (5)
O12	0.0148 (5)	0.0136 (5)	0.0292 (7)	−0.0051 (5)	−0.0077 (5)	0.0010 (5)
O13	0.0268 (7)	0.0164 (6)	0.0403 (9)	−0.0028 (6)	−0.0073 (6)	−0.0010 (6)
O14	0.0189 (7)	0.0288 (8)	0.0527 (10)	0.0016 (6)	−0.0157 (7)	−0.0049 (7)
O15	0.0135 (5)	0.0126 (5)	0.0245 (6)	−0.0029 (4)	−0.0040 (5)	−0.0013 (5)
O16	0.0149 (6)	0.0186 (6)	0.0253 (6)	−0.0057 (5)	−0.0052 (5)	−0.0030 (5)
O17	0.0296 (8)	0.0225 (7)	0.0483 (9)	−0.0140 (6)	−0.0121 (7)	−0.0035 (6)
O18	0.0198 (7)	0.0330 (9)	0.0632 (11)	−0.0131 (6)	−0.0149 (7)	−0.0009 (8)
N1	0.0182 (7)	0.0172 (7)	0.0284 (8)	−0.0021 (6)	−0.0064 (6)	−0.0019 (6)
N2	0.0176 (7)	0.0142 (7)	0.0353 (9)	−0.0083 (6)	−0.0099 (6)	0.0012 (6)
N3	0.0179 (7)	0.0217 (8)	0.0257 (8)	−0.0104 (6)	−0.0060 (6)	0.0025 (6)
N4	0.0228 (8)	0.0132 (7)	0.0401 (10)	−0.0026 (6)	−0.0136 (7)	−0.0065 (7)
N5	0.0187 (7)	0.0214 (8)	0.0262 (8)	−0.0006 (6)	−0.0086 (6)	−0.0039 (6)
N6	0.0181 (7)	0.0163 (7)	0.0325 (9)	−0.0092 (6)	−0.0075 (6)	−0.0002 (6)
N7	0.0213 (8)	0.0211 (8)	0.0379 (9)	−0.0120 (7)	−0.0109 (7)	0.0047 (7)
N8	0.0176 (7)	0.0150 (7)	0.0351 (9)	−0.0025 (6)	−0.0075 (7)	−0.0068 (6)
C1	0.0143 (7)	0.0143 (7)	0.0312 (9)	−0.0056 (6)	−0.0076 (7)	−0.0013 (7)
C2	0.0132 (7)	0.0138 (7)	0.0206 (8)	−0.0055 (6)	−0.0040 (6)	−0.0023 (6)
C3	0.0155 (7)	0.0133 (7)	0.0222 (8)	−0.0054 (6)	−0.0058 (6)	−0.0021 (6)
C4	0.0151 (7)	0.0149 (7)	0.0225 (8)	−0.0026 (6)	−0.0062 (6)	−0.0017 (6)
C5	0.0142 (7)	0.0215 (8)	0.0284 (9)	−0.0075 (7)	−0.0081 (7)	0.0007 (7)
C6	0.0164 (8)	0.0193 (8)	0.0274 (9)	−0.0099 (7)	−0.0069 (7)	−0.0002 (7)
C7	0.0144 (7)	0.0163 (7)	0.0191 (8)	−0.0067 (6)	−0.0040 (6)	−0.0011 (6)
C8	0.0143 (7)	0.0135 (7)	0.0253 (8)	−0.0031 (6)	−0.0058 (6)	−0.0018 (6)
C9	0.0146 (7)	0.0119 (7)	0.0207 (8)	−0.0020 (6)	−0.0050 (6)	−0.0018 (6)
C10	0.0155 (7)	0.0135 (7)	0.0198 (8)	−0.0056 (6)	−0.0040 (6)	0.0002 (6)
C11	0.0153 (7)	0.0175 (8)	0.0206 (8)	−0.0067 (6)	−0.0047 (6)	0.0012 (6)
C12	0.0150 (8)	0.0193 (8)	0.0266 (9)	−0.0031 (7)	−0.0060 (7)	−0.0002 (7)
C13	0.0189 (8)	0.0143 (8)	0.0299 (9)	−0.0008 (7)	−0.0076 (7)	−0.0031 (7)
C14	0.0195 (8)	0.0123 (7)	0.0230 (8)	−0.0037 (6)	−0.0076 (6)	−0.0013 (6)
C15	0.0123 (7)	0.0147 (7)	0.0201 (8)	−0.0051 (6)	−0.0045 (6)	−0.0003 (6)
C16	0.0129 (7)	0.0151 (7)	0.0190 (7)	−0.0057 (6)	−0.0046 (6)	−0.0012 (6)
C17	0.0153 (7)	0.0149 (7)	0.0194 (8)	−0.0054 (6)	−0.0051 (6)	−0.0016 (6)
C18	0.0152 (7)	0.0176 (8)	0.0230 (8)	−0.0027 (6)	−0.0066 (6)	−0.0021 (6)
C19	0.0143 (7)	0.0263 (9)	0.0260 (9)	−0.0068 (7)	−0.0067 (7)	−0.0033 (7)
C20	0.0157 (8)	0.0220 (8)	0.0258 (9)	−0.0092 (7)	−0.0048 (6)	−0.0031 (7)
C21	0.0144 (7)	0.0182 (8)	0.0183 (7)	−0.0074 (6)	−0.0030 (6)	−0.0031 (6)
C22	0.0132 (7)	0.0140 (7)	0.0179 (7)	−0.0038 (6)	−0.0033 (6)	0.0013 (6)
C23	0.0135 (7)	0.0129 (7)	0.0191 (7)	−0.0044 (6)	−0.0045 (6)	0.0014 (6)
C24	0.0177 (8)	0.0135 (7)	0.0217 (8)	−0.0065 (6)	−0.0046 (6)	0.0020 (6)
C25	0.0171 (8)	0.0171 (8)	0.0261 (9)	−0.0086 (6)	−0.0076 (7)	0.0021 (7)
C26	0.0142 (8)	0.0203 (8)	0.0308 (10)	−0.0054 (7)	−0.0047 (7)	0.0009 (7)
C27	0.0161 (8)	0.0162 (8)	0.0300 (9)	−0.0032 (6)	−0.0046 (7)	−0.0039 (7)
C28	0.0148 (7)	0.0140 (7)	0.0214 (8)	−0.0038 (6)	−0.0040 (6)	−0.0010 (6)
C29	0.0180 (8)	0.0146 (7)	0.0261 (9)	−0.0065 (6)	−0.0077 (7)	−0.0004 (6)
C30	0.0196 (8)	0.0151 (8)	0.0262 (9)	−0.0070 (7)	−0.0070 (7)	0.0005 (6)
C31	0.0262 (9)	0.0182 (8)	0.0233 (9)	−0.0082 (7)	−0.0060 (7)	0.0005 (7)
C32	0.0332 (11)	0.0211 (9)	0.0283 (10)	−0.0042 (8)	−0.0045 (8)	0.0035 (8)
C33	0.0161 (17)	0.014 (2)	0.021 (3)	−0.0074 (17)	−0.002 (2)	0.0000 (17)
C34	0.0210 (16)	0.0211 (17)	0.0200 (17)	−0.0113 (14)	−0.0024 (13)	−0.0035 (13)
C35	0.0237 (18)	0.024 (2)	0.0185 (17)	−0.0107 (16)	−0.0014 (13)	−0.0003 (15)
C36	0.048 (3)	0.025 (2)	0.027 (2)	−0.015 (2)	−0.0102 (19)	0.0028 (17)
C33X	0.0125 (17)	0.015 (2)	0.027 (3)	−0.0027 (17)	0.000 (2)	−0.0018 (19)
C34X	0.0197 (17)	0.0153 (16)	0.0261 (19)	−0.0076 (14)	−0.0041 (14)	0.0003 (13)
C35X	0.029 (2)	0.023 (2)	0.024 (2)	−0.0128 (19)	−0.0037 (16)	−0.0020 (17)
C36X	0.042 (3)	0.029 (2)	0.023 (2)	−0.018 (2)	−0.0073 (18)	0.0080 (17)
C37	0.0159 (7)	0.0184 (8)	0.0232 (8)	−0.0077 (6)	−0.0038 (6)	0.0009 (6)
C38	0.0261 (9)	0.0242 (9)	0.0258 (9)	−0.0123 (8)	−0.0068 (7)	−0.0006 (7)
C39	0.0259 (9)	0.0293 (10)	0.0224 (9)	−0.0108 (8)	−0.0044 (7)	−0.0011 (7)
C40	0.0320 (11)	0.0278 (10)	0.0268 (10)	−0.0112 (9)	−0.0062 (8)	0.0028 (8)
C41	0.0183 (8)	0.0154 (8)	0.0258 (9)	−0.0079 (6)	−0.0028 (7)	0.0000 (6)
C42	0.0213 (9)	0.0173 (8)	0.0274 (9)	−0.0047 (7)	−0.0040 (7)	0.0051 (7)
C43	0.0295 (10)	0.0203 (9)	0.0247 (9)	−0.0088 (8)	−0.0053 (8)	0.0034 (7)
C44	0.0478 (14)	0.0263 (11)	0.0368 (12)	−0.0204 (10)	−0.0106 (10)	0.0049 (9)
C45	0.0204 (8)	0.0138 (8)	0.0357 (10)	−0.0073 (7)	−0.0064 (7)	−0.0011 (7)
C46	0.0223 (9)	0.0145 (8)	0.0440 (12)	−0.0087 (7)	−0.0133 (8)	0.0014 (8)
C47	0.0264 (10)	0.0191 (9)	0.0417 (12)	−0.0128 (8)	−0.0005 (9)	−0.0051 (8)
C48	0.0464 (14)	0.0229 (10)	0.0499 (14)	−0.0176 (10)	−0.0225 (11)	0.0072 (10)
C49	0.0230 (11)	0.0272 (12)	0.0259 (11)	−0.0132 (10)	−0.0089 (9)	0.0069 (10)
C50	0.0261 (12)	0.0307 (13)	0.0248 (11)	−0.0045 (10)	−0.0083 (9)	−0.0031 (9)
C51	0.0390 (14)	0.0191 (10)	0.0268 (13)	−0.0118 (10)	−0.0105 (10)	−0.0019 (9)
C52	0.0288 (13)	0.0296 (13)	0.0300 (14)	−0.0127 (10)	−0.0084 (12)	−0.0011 (12)
C53	0.0228 (9)	0.0287 (10)	0.0202 (8)	−0.0104 (8)	−0.0058 (7)	0.0043 (7)
C54	0.0264 (10)	0.0369 (11)	0.0170 (8)	−0.0103 (9)	−0.0048 (7)	−0.0025 (8)
C55	0.0274 (10)	0.0309 (11)	0.0320 (11)	−0.0074 (9)	−0.0096 (8)	−0.0086 (9)
C56	0.0389 (13)	0.0441 (14)	0.0408 (13)	−0.0135 (11)	−0.0174 (11)	−0.0114 (11)
C57	0.0194 (8)	0.0172 (8)	0.0222 (8)	−0.0086 (7)	−0.0033 (6)	0.0021 (6)
C58	0.0232 (8)	0.0140 (7)	0.0219 (8)	−0.0090 (7)	−0.0060 (7)	0.0019 (6)
C59	0.0355 (11)	0.0275 (10)	0.0287 (10)	−0.0159 (9)	−0.0051 (9)	0.0087 (8)
C60	0.0564 (15)	0.0239 (10)	0.0293 (11)	−0.0204 (10)	−0.0145 (10)	0.0088 (8)

### Geometric parameters (Å, °)

**Table:**

Sn1—O1	2.0436 (12)	C33—H33A	0.9900
Sn1—C33	2.050 (7)	C33—H33B	0.9900
Sn1—C29	2.1234 (18)	C34—C35	1.541 (5)
Sn1—O2	2.1445 (12)	C34—H34A	0.9900
Sn1—C33X	2.232 (7)	C34—H34B	0.9900
Sn1—O4	2.2643 (14)	C35—C36	1.516 (6)
Sn1—Sn2	3.29699 (17)	C35—H35A	0.9900
Sn2—O2	2.0474 (12)	C35—H35B	0.9900
Sn2—C41	2.1343 (18)	C36—H36A	0.9800
Sn2—C37	2.1351 (17)	C36—H36B	0.9800
Sn2—O1	2.1504 (12)	C36—H36C	0.9800
Sn2—O11	2.2583 (13)	C33X—C34X	1.525 (8)
Sn3—C49X	1.861 (16)	C33X—H33C	0.9900
Sn3—O1	2.0384 (12)	C33X—H33D	0.9900
Sn3—C45	2.1283 (19)	C34X—C35X	1.527 (6)
Sn3—C49	2.176 (3)	C34X—H34C	0.9900
Sn3—O7	2.1992 (12)	C34X—H34D	0.9900
Sn3—O3	2.3178 (13)	C35X—C36X	1.526 (6)
Sn4—O2	2.0439 (12)	C35X—H35C	0.9900
Sn4—C53	2.1293 (19)	C35X—H35D	0.9900
Sn4—C57	2.1370 (17)	C36X—H36D	0.9800
Sn4—O15	2.1908 (12)	C36X—H36E	0.9800
Sn4—O12	2.3115 (12)	C36X—H36F	0.9800
O3—C1	1.270 (2)	C37—C38	1.524 (3)
O4—C1	1.263 (2)	C37—H37A	0.9900
O5—N1	1.233 (2)	C37—H37B	0.9900
O6—N1	1.237 (2)	C38—C39	1.530 (3)
O7—C8	1.297 (2)	C38—H38A	0.9900
O8—C8	1.250 (2)	C38—H38B	0.9900
O9—N3	1.243 (2)	C39—C40	1.525 (3)
O10—N3	1.234 (2)	C39—H39A	0.9900
O11—C15	1.265 (2)	C39—H39B	0.9900
O12—C15	1.268 (2)	C40—H40A	0.9800
O13—N5	1.241 (2)	C40—H40B	0.9800
O14—N5	1.232 (2)	C40—H40C	0.9800
O15—C22	1.301 (2)	C41—C42	1.539 (3)
O16—C22	1.243 (2)	C41—H41A	0.9900
O17—N7	1.243 (2)	C41—H41B	0.9900
O18—N7	1.233 (2)	C42—C43	1.531 (3)
N1—C4	1.444 (2)	C42—H42A	0.9900
N2—C7	1.346 (2)	C42—H42B	0.9900
N2—H1N2	0.85 (3)	C43—C44	1.519 (3)
N2—H2N2	0.83 (2)	C43—H43A	0.9900
N3—C11	1.437 (2)	C43—H43B	0.9900
N4—C14	1.341 (2)	C44—H44A	0.9800
N4—H1N4	0.85 (3)	C44—H44B	0.9800
N4—H2N4	0.87 (3)	C44—H44C	0.9800
N5—C18	1.440 (2)	C45—C46	1.537 (3)
N6—C21	1.341 (2)	C45—H45A	0.9900
N6—H1N6	0.82 (3)	C45—H45B	0.9900
N6—H2N6	0.83 (2)	C46—C47	1.527 (3)
N7—C25	1.439 (2)	C46—H46A	0.9900
N8—C28	1.340 (2)	C46—H46B	0.9900
N8—H1N8	0.86 (3)	C47—C48	1.517 (3)
N8—H2N8	0.82 (2)	C47—H47A	0.9900
C1—C2	1.490 (2)	C47—H47B	0.9900
C2—C3	1.389 (2)	C48—H48A	0.9800
C2—C7	1.427 (2)	C48—H48B	0.9800
C3—C4	1.380 (2)	C48—H48C	0.9800
C3—H3A	0.9500	C49—C50	1.529 (4)
C4—C5	1.399 (3)	C49—H49A	0.9900
C5—C6	1.367 (3)	C49—H49C	0.9900
C5—H5A	0.9500	C50—C51	1.524 (4)
C6—C7	1.417 (2)	C50—H50A	0.9900
C6—H6A	0.9500	C50—H50B	0.9900
C8—C9	1.481 (2)	C51—C52	1.522 (4)
C9—C10	1.390 (2)	C51—H51A	0.9900
C9—C14	1.430 (2)	C51—H51B	0.9900
C10—C11	1.385 (2)	C52—H52A	0.9800
C10—H10A	0.9500	C52—H52B	0.9800
C11—C12	1.398 (3)	C52—H52C	0.9800
C12—C13	1.368 (3)	C49X—C50X	1.55 (2)
C12—H12A	0.9500	C49X—H49B	0.9900
C13—C14	1.424 (3)	C49X—H49D	0.9900
C13—H13A	0.9500	C50X—C51X	1.46 (2)
C15—C16	1.488 (2)	C50X—H50C	0.9900
C16—C17	1.394 (2)	C50X—H50D	0.9900
C16—C21	1.428 (2)	C51X—C52X	1.52 (3)
C17—C18	1.377 (2)	C51X—H51C	0.9900
C17—H17A	0.9500	C51X—H51D	0.9900
C18—C19	1.400 (3)	C52X—H52D	0.9800
C19—C20	1.367 (3)	C52X—H52E	0.9800
C19—H19A	0.9500	C52X—H52F	0.9800
C20—C21	1.423 (2)	C53—C54	1.533 (3)
C20—H20A	0.9500	C53—H53A	0.9900
C22—C23	1.487 (2)	C53—H53B	0.9900
C23—C24	1.391 (2)	C54—C55	1.522 (3)
C23—C28	1.427 (2)	C54—H54A	0.9900
C24—C25	1.380 (2)	C54—H54B	0.9900
C24—H24A	0.9500	C55—C56	1.525 (3)
C25—C26	1.400 (3)	C55—H55A	0.9900
C26—C27	1.366 (3)	C55—H55B	0.9900
C26—H26A	0.9500	C56—H56A	0.9800
C27—C28	1.424 (2)	C56—H56B	0.9800
C27—H27A	0.9500	C56—H56C	0.9800
C29—C30	1.527 (2)	C57—C58	1.523 (2)
C29—H29A	0.9900	C57—H57A	0.9900
C29—H29B	0.9900	C57—H57B	0.9900
C30—C31	1.530 (3)	C58—C59	1.527 (3)
C30—H30A	0.9900	C58—H58A	0.9900
C30—H30B	0.9900	C58—H58B	0.9900
C31—C32	1.525 (3)	C59—C60	1.528 (3)
C31—H31A	0.9900	C59—H59A	0.9900
C31—H31B	0.9900	C59—H59B	0.9900
C32—H32A	0.9800	C60—H60A	0.9800
C32—H32B	0.9800	C60—H60B	0.9800
C32—H32C	0.9800	C60—H60C	0.9800
C33—C34	1.526 (6)		
			
O1—Sn1—C33	102.26 (19)	C34—C33—Sn1	116.7 (4)
O1—Sn1—C29	109.79 (6)	C34—C33—H33A	108.1
C33—Sn1—C29	144.4 (2)	Sn1—C33—H33A	108.1
O1—Sn1—O2	76.44 (5)	C34—C33—H33B	108.1
C33—Sn1—O2	104.49 (17)	Sn1—C33—H33B	108.1
C29—Sn1—O2	97.91 (6)	H33A—C33—H33B	107.3
O1—Sn1—C33X	106.93 (18)	C33—C34—C35	112.0 (4)
C33—Sn1—C33X	11.9 (2)	C33—C34—H34A	109.2
C29—Sn1—C33X	143.02 (19)	C35—C34—H34A	109.2
O2—Sn1—C33X	94.69 (18)	C33—C34—H34B	109.2
O1—Sn1—O4	89.08 (5)	C35—C34—H34B	109.2
C33—Sn1—O4	82.72 (16)	H34A—C34—H34B	107.9
C29—Sn1—O4	82.63 (6)	C36—C35—C34	113.9 (3)
O2—Sn1—O4	164.84 (5)	C36—C35—H35A	108.8
C33X—Sn1—O4	93.80 (17)	C34—C35—H35A	108.8
O1—Sn1—Sn2	39.34 (3)	C36—C35—H35B	108.8
C33—Sn1—Sn2	107.5 (2)	C34—C35—H35B	108.8
C29—Sn1—Sn2	107.14 (5)	H35A—C35—H35B	107.7
O2—Sn1—Sn2	37.11 (3)	C34X—C33X—Sn1	111.2 (4)
C33X—Sn1—Sn2	103.85 (19)	C34X—C33X—H33C	109.4
O4—Sn1—Sn2	128.23 (4)	Sn1—C33X—H33C	109.4
O2—Sn2—C41	116.69 (6)	C34X—C33X—H33D	109.4
O2—Sn2—C37	102.20 (6)	Sn1—C33X—H33D	109.4
C41—Sn2—C37	139.14 (7)	H33C—C33X—H33D	108.0
O2—Sn2—O1	76.23 (5)	C33X—C34X—C35X	113.1 (4)
C41—Sn2—O1	97.59 (6)	C33X—C34X—H34C	109.0
C37—Sn2—O1	103.59 (6)	C35X—C34X—H34C	109.0
O2—Sn2—O11	87.60 (5)	C33X—C34X—H34D	109.0
C41—Sn2—O11	83.03 (6)	C35X—C34X—H34D	109.0
C37—Sn2—O11	86.75 (6)	H34C—C34X—H34D	107.8
O1—Sn2—O11	162.25 (5)	C36X—C35X—C34X	112.2 (4)
O2—Sn2—Sn1	39.19 (3)	C36X—C35X—H35C	109.2
C41—Sn2—Sn1	111.68 (5)	C34X—C35X—H35C	109.2
C37—Sn2—Sn1	106.18 (5)	C36X—C35X—H35D	109.2
O1—Sn2—Sn1	37.04 (3)	C34X—C35X—H35D	109.2
O11—Sn2—Sn1	126.47 (3)	H35C—C35X—H35D	107.9
C49X—Sn3—O1	117.0 (5)	C35X—C36X—H36D	109.5
C49X—Sn3—C45	128.7 (6)	C35X—C36X—H36E	109.5
O1—Sn3—C45	112.74 (7)	H36D—C36X—H36E	109.5
C49X—Sn3—C49	14.3 (5)	C35X—C36X—H36F	109.5
O1—Sn3—C49	103.90 (8)	H36D—C36X—H36F	109.5
C45—Sn3—C49	142.79 (9)	H36E—C36X—H36F	109.5
C49X—Sn3—O7	105.0 (5)	C38—C37—Sn2	118.39 (13)
O1—Sn3—O7	79.83 (5)	C38—C37—H37A	107.7
C45—Sn3—O7	94.91 (6)	Sn2—C37—H37A	107.7
C49—Sn3—O7	97.73 (7)	C38—C37—H37B	107.7
C49X—Sn3—O3	85.3 (5)	Sn2—C37—H37B	107.7
O1—Sn3—O3	90.46 (5)	H37A—C37—H37B	107.1
C45—Sn3—O3	82.56 (6)	C37—C38—C39	112.21 (16)
C49—Sn3—O3	91.11 (7)	C37—C38—H38A	109.2
O7—Sn3—O3	168.18 (5)	C39—C38—H38A	109.2
O2—Sn4—C53	105.16 (7)	C37—C38—H38B	109.2
O2—Sn4—C57	112.18 (6)	C39—C38—H38B	109.2
C53—Sn4—C57	140.54 (8)	H38A—C38—H38B	107.9
O2—Sn4—O15	80.07 (5)	C40—C39—C38	113.81 (17)
C53—Sn4—O15	99.69 (6)	C40—C39—H39A	108.8
C57—Sn4—O15	98.85 (6)	C38—C39—H39A	108.8
O2—Sn4—O12	89.13 (5)	C40—C39—H39B	108.8
C53—Sn4—O12	89.47 (6)	C38—C39—H39B	108.8
C57—Sn4—O12	79.01 (6)	H39A—C39—H39B	107.7
O15—Sn4—O12	167.33 (5)	C39—C40—H40A	109.5
Sn3—O1—Sn1	133.09 (6)	C39—C40—H40B	109.5
Sn3—O1—Sn2	123.16 (6)	H40A—C40—H40B	109.5
Sn1—O1—Sn2	103.62 (5)	C39—C40—H40C	109.5
Sn4—O2—Sn2	133.50 (6)	H40A—C40—H40C	109.5
Sn4—O2—Sn1	121.48 (6)	H40B—C40—H40C	109.5
Sn2—O2—Sn1	103.70 (5)	C42—C41—Sn2	112.73 (12)
C1—O3—Sn3	127.34 (12)	C42—C41—H41A	109.0
C1—O4—Sn1	130.17 (13)	Sn2—C41—H41A	109.0
C8—O7—Sn3	105.06 (11)	C42—C41—H41B	109.0
C15—O11—Sn2	124.96 (11)	Sn2—C41—H41B	109.0
C15—O12—Sn4	128.32 (11)	H41A—C41—H41B	107.8
C22—O15—Sn4	105.22 (11)	C43—C42—C41	113.99 (16)
O5—N1—O6	122.51 (16)	C43—C42—H42A	108.8
O5—N1—C4	118.98 (17)	C41—C42—H42A	108.8
O6—N1—C4	118.51 (15)	C43—C42—H42B	108.8
C7—N2—H1N2	117.9 (18)	C41—C42—H42B	108.8
C7—N2—H2N2	116.8 (17)	H42A—C42—H42B	107.6
H1N2—N2—H2N2	123 (2)	C44—C43—C42	113.81 (18)
O10—N3—O9	122.07 (17)	C44—C43—H43A	108.8
O10—N3—C11	119.24 (16)	C42—C43—H43A	108.8
O9—N3—C11	118.69 (15)	C44—C43—H43B	108.8
C14—N4—H1N4	121.5 (19)	C42—C43—H43B	108.8
C14—N4—H2N4	119.3 (18)	H43A—C43—H43B	107.7
H1N4—N4—H2N4	119 (3)	C43—C44—H44A	109.5
O14—N5—O13	122.57 (17)	C43—C44—H44B	109.5
O14—N5—C18	118.92 (18)	H44A—C44—H44B	109.5
O13—N5—C18	118.51 (16)	C43—C44—H44C	109.5
C21—N6—H1N6	119.1 (19)	H44A—C44—H44C	109.5
C21—N6—H2N6	119.1 (16)	H44B—C44—H44C	109.5
H1N6—N6—H2N6	122 (2)	C46—C45—Sn3	112.88 (14)
O18—N7—O17	121.85 (18)	C46—C45—H45A	109.0
O18—N7—C25	119.37 (17)	Sn3—C45—H45A	109.0
O17—N7—C25	118.78 (16)	C46—C45—H45B	109.0
C28—N8—H1N8	119 (2)	Sn3—C45—H45B	109.0
C28—N8—H2N8	119.4 (17)	H45A—C45—H45B	107.8
H1N8—N8—H2N8	118 (3)	C47—C46—C45	113.72 (18)
O4—C1—O3	123.22 (16)	C47—C46—H46A	108.8
O4—C1—C2	117.46 (15)	C45—C46—H46A	108.8
O3—C1—C2	119.32 (16)	C47—C46—H46B	108.8
C3—C2—C7	119.34 (15)	C45—C46—H46B	108.8
C3—C2—C1	117.60 (15)	H46A—C46—H46B	107.7
C7—C2—C1	123.00 (15)	C48—C47—C46	113.82 (18)
C4—C3—C2	120.50 (16)	C48—C47—H47A	108.8
C4—C3—H3A	119.8	C46—C47—H47A	108.8
C2—C3—H3A	119.8	C48—C47—H47B	108.8
C3—C4—C5	121.30 (16)	C46—C47—H47B	108.8
C3—C4—N1	119.43 (16)	H47A—C47—H47B	107.7
C5—C4—N1	119.22 (16)	C47—C48—H48A	109.5
C6—C5—C4	118.96 (16)	C47—C48—H48B	109.5
C6—C5—H5A	120.5	H48A—C48—H48B	109.5
C4—C5—H5A	120.5	C47—C48—H48C	109.5
C5—C6—C7	121.66 (17)	H48A—C48—H48C	109.5
C5—C6—H6A	119.2	H48B—C48—H48C	109.5
C7—C6—H6A	119.2	C50—C49—Sn3	116.96 (18)
N2—C7—C6	119.03 (16)	C50—C49—H49A	108.1
N2—C7—C2	122.75 (16)	Sn3—C49—H49A	108.1
C6—C7—C2	118.22 (15)	C50—C49—H49C	108.1
O8—C8—O7	120.61 (16)	Sn3—C49—H49C	108.1
O8—C8—C9	121.48 (16)	H49A—C49—H49C	107.3
O7—C8—C9	117.90 (16)	C51—C50—C49	115.3 (2)
C10—C9—C14	119.38 (16)	C51—C50—H50A	108.4
C10—C9—C8	119.00 (15)	C49—C50—H50A	108.4
C14—C9—C8	121.61 (16)	C51—C50—H50B	108.4
C11—C10—C9	120.51 (16)	C49—C50—H50B	108.4
C11—C10—H10A	119.7	H50A—C50—H50B	107.5
C9—C10—H10A	119.7	C52—C51—C50	114.2 (2)
C10—C11—C12	121.17 (17)	C52—C51—H51A	108.7
C10—C11—N3	119.22 (16)	C50—C51—H51A	108.7
C12—C11—N3	119.61 (16)	C52—C51—H51B	108.7
C13—C12—C11	119.26 (17)	C50—C51—H51B	108.7
C13—C12—H12A	120.4	H51A—C51—H51B	107.6
C11—C12—H12A	120.4	C50X—C49X—Sn3	115.6 (12)
C12—C13—C14	121.54 (17)	C50X—C49X—H49B	108.4
C12—C13—H13A	119.2	Sn3—C49X—H49B	108.4
C14—C13—H13A	119.2	C50X—C49X—H49D	108.4
N4—C14—C13	119.67 (16)	Sn3—C49X—H49D	108.4
N4—C14—C9	122.22 (17)	H49B—C49X—H49D	107.4
C13—C14—C9	118.10 (17)	C51X—C50X—C49X	118.2 (14)
O11—C15—O12	123.40 (15)	C51X—C50X—H50C	107.8
O11—C15—C16	117.83 (15)	C49X—C50X—H50C	107.8
O12—C15—C16	118.76 (15)	C51X—C50X—H50D	107.8
C17—C16—C21	119.43 (15)	C49X—C50X—H50D	107.8
C17—C16—C15	117.28 (15)	H50C—C50X—H50D	107.1
C21—C16—C15	123.23 (15)	C50X—C51X—C52X	113.3 (15)
C18—C17—C16	120.49 (16)	C50X—C51X—H51C	108.9
C18—C17—H17A	119.8	C52X—C51X—H51C	108.9
C16—C17—H17A	119.8	C50X—C51X—H51D	108.9
C17—C18—C19	121.23 (17)	C52X—C51X—H51D	108.9
C17—C18—N5	119.42 (17)	H51C—C51X—H51D	107.7
C19—C18—N5	119.35 (16)	C51X—C52X—H52D	109.5
C20—C19—C18	119.31 (17)	C51X—C52X—H52E	109.5
C20—C19—H19A	120.3	H52D—C52X—H52E	109.5
C18—C19—H19A	120.3	C51X—C52X—H52F	109.5
C19—C20—C21	121.41 (17)	H52D—C52X—H52F	109.5
C19—C20—H20A	119.3	H52E—C52X—H52F	109.5
C21—C20—H20A	119.3	C54—C53—Sn4	115.86 (13)
N6—C21—C20	118.95 (16)	C54—C53—H53A	108.3
N6—C21—C16	122.97 (16)	Sn4—C53—H53A	108.3
C20—C21—C16	118.07 (16)	C54—C53—H53B	108.3
O16—C22—O15	120.67 (15)	Sn4—C53—H53B	108.3
O16—C22—C23	121.77 (16)	H53A—C53—H53B	107.4
O15—C22—C23	117.55 (15)	C55—C54—C53	114.43 (17)
C24—C23—C28	119.26 (16)	C55—C54—H54A	108.7
C24—C23—C22	119.27 (15)	C53—C54—H54A	108.7
C28—C23—C22	121.45 (16)	C55—C54—H54B	108.7
C25—C24—C23	120.59 (16)	C53—C54—H54B	108.7
C25—C24—H24A	119.7	H54A—C54—H54B	107.6
C23—C24—H24A	119.7	C54—C55—C56	113.45 (19)
C24—C25—C26	121.30 (17)	C54—C55—H55A	108.9
C24—C25—N7	119.29 (17)	C56—C55—H55A	108.9
C26—C25—N7	119.41 (16)	C54—C55—H55B	108.9
C27—C26—C25	119.04 (17)	C56—C55—H55B	108.9
C27—C26—H26A	120.5	H55A—C55—H55B	107.7
C25—C26—H26A	120.5	C55—C56—H56A	109.5
C26—C27—C28	121.58 (17)	C55—C56—H56B	109.5
C26—C27—H27A	119.2	H56A—C56—H56B	109.5
C28—C27—H27A	119.2	C55—C56—H56C	109.5
N8—C28—C27	119.23 (16)	H56A—C56—H56C	109.5
N8—C28—C23	122.54 (16)	H56B—C56—H56C	109.5
C27—C28—C23	118.22 (16)	C58—C57—Sn4	119.96 (12)
C30—C29—Sn1	115.88 (12)	C58—C57—H57A	107.3
C30—C29—H29A	108.3	Sn4—C57—H57A	107.3
Sn1—C29—H29A	108.3	C58—C57—H57B	107.3
C30—C29—H29B	108.3	Sn4—C57—H57B	107.3
Sn1—C29—H29B	108.3	H57A—C57—H57B	106.9
H29A—C29—H29B	107.4	C57—C58—C59	112.36 (16)
C29—C30—C31	112.29 (15)	C57—C58—H58A	109.1
C29—C30—H30A	109.1	C59—C58—H58A	109.1
C31—C30—H30A	109.1	C57—C58—H58B	109.1
C29—C30—H30B	109.1	C59—C58—H58B	109.1
C31—C30—H30B	109.1	H58A—C58—H58B	107.9
H30A—C30—H30B	107.9	C58—C59—C60	113.17 (19)
C32—C31—C30	112.46 (16)	C58—C59—H59A	108.9
C32—C31—H31A	109.1	C60—C59—H59A	108.9
C30—C31—H31A	109.1	C58—C59—H59B	108.9
C32—C31—H31B	109.1	C60—C59—H59B	108.9
C30—C31—H31B	109.1	H59A—C59—H59B	107.8
H31A—C31—H31B	107.8	C59—C60—H60A	109.5
C31—C32—H32A	109.5	C59—C60—H60B	109.5
C31—C32—H32B	109.5	H60A—C60—H60B	109.5
H32A—C32—H32B	109.5	C59—C60—H60C	109.5
C31—C32—H32C	109.5	H60A—C60—H60C	109.5
H32A—C32—H32C	109.5	H60B—C60—H60C	109.5
H32B—C32—H32C	109.5		
			
O1—Sn1—Sn2—O2	−179.19 (8)	Sn3—O7—C8—C9	−178.59 (13)
C33—Sn1—Sn2—O2	−91.07 (16)	O8—C8—C9—C10	171.89 (17)
C29—Sn1—Sn2—O2	80.28 (8)	O7—C8—C9—C10	−8.3 (3)
C33X—Sn1—Sn2—O2	−79.27 (17)	O8—C8—C9—C14	−7.8 (3)
O4—Sn1—Sn2—O2	174.41 (8)	O7—C8—C9—C14	172.08 (16)
O1—Sn1—Sn2—C41	−73.09 (8)	C14—C9—C10—C11	−0.1 (3)
C33—Sn1—Sn2—C41	15.03 (16)	C8—C9—C10—C11	−179.78 (16)
C29—Sn1—Sn2—C41	−173.62 (8)	C9—C10—C11—C12	1.3 (3)
O2—Sn1—Sn2—C41	106.10 (8)	C9—C10—C11—N3	−179.12 (16)
C33X—Sn1—Sn2—C41	26.83 (17)	O10—N3—C11—C10	178.31 (17)
O4—Sn1—Sn2—C41	−79.49 (8)	O9—N3—C11—C10	−2.3 (3)
O1—Sn1—Sn2—C37	91.25 (8)	O10—N3—C11—C12	−2.1 (3)
C33—Sn1—Sn2—C37	179.37 (16)	O9—N3—C11—C12	177.28 (17)
C29—Sn1—Sn2—C37	−9.28 (7)	C10—C11—C12—C13	−0.9 (3)
O2—Sn1—Sn2—C37	−89.56 (8)	N3—C11—C12—C13	179.52 (17)
C33X—Sn1—Sn2—C37	−168.83 (17)	C11—C12—C13—C14	−0.7 (3)
O4—Sn1—Sn2—C37	84.85 (7)	C12—C13—C14—N4	−177.46 (19)
C33—Sn1—Sn2—O1	88.12 (16)	C12—C13—C14—C9	1.8 (3)
C29—Sn1—Sn2—O1	−100.53 (8)	C10—C9—C14—N4	177.86 (18)
O2—Sn1—Sn2—O1	179.19 (8)	C8—C9—C14—N4	−2.5 (3)
C33X—Sn1—Sn2—O1	99.92 (17)	C10—C9—C14—C13	−1.4 (3)
O4—Sn1—Sn2—O1	−6.40 (8)	C8—C9—C14—C13	178.28 (17)
O1—Sn1—Sn2—O11	−170.64 (7)	Sn2—O11—C15—O12	46.4 (2)
C33—Sn1—Sn2—O11	−82.52 (16)	Sn2—O11—C15—C16	−132.19 (13)
C29—Sn1—Sn2—O11	88.83 (7)	Sn4—O12—C15—O11	18.1 (3)
O2—Sn1—Sn2—O11	8.55 (7)	Sn4—O12—C15—C16	−163.34 (11)
C33X—Sn1—Sn2—O11	−70.72 (16)	O11—C15—C16—C17	8.0 (2)
O4—Sn1—Sn2—O11	−177.04 (6)	O12—C15—C16—C17	−170.66 (16)
C49X—Sn3—O1—Sn1	67.5 (6)	O11—C15—C16—C21	−174.90 (16)
C45—Sn3—O1—Sn1	−99.67 (10)	O12—C15—C16—C21	6.5 (3)
C49—Sn3—O1—Sn1	73.72 (11)	C21—C16—C17—C18	−0.1 (3)
O7—Sn3—O1—Sn1	169.24 (10)	C15—C16—C17—C18	177.10 (16)
O3—Sn3—O1—Sn1	−17.54 (10)	C16—C17—C18—C19	0.8 (3)
C49X—Sn3—O1—Sn2	−117.4 (6)	C16—C17—C18—N5	−179.03 (16)
C45—Sn3—O1—Sn2	75.44 (9)	O14—N5—C18—C17	170.25 (18)
C49—Sn3—O1—Sn2	−111.18 (9)	O13—N5—C18—C17	−9.9 (3)
O7—Sn3—O1—Sn2	−15.65 (7)	O14—N5—C18—C19	−9.6 (3)
O3—Sn3—O1—Sn2	157.57 (8)	O13—N5—C18—C19	170.24 (18)
C33—Sn1—O1—Sn3	73.10 (19)	C17—C18—C19—C20	0.3 (3)
C29—Sn1—O1—Sn3	−91.06 (10)	N5—C18—C19—C20	−179.79 (17)
O2—Sn1—O1—Sn3	175.28 (11)	C18—C19—C20—C21	−2.2 (3)
C33X—Sn1—O1—Sn3	84.4 (2)	C19—C20—C21—N6	−176.64 (18)
O4—Sn1—O1—Sn3	−9.24 (10)	C19—C20—C21—C16	2.9 (3)
Sn2—Sn1—O1—Sn3	175.78 (13)	C17—C16—C21—N6	177.84 (17)
C33—Sn1—O1—Sn2	−102.68 (17)	C15—C16—C21—N6	0.8 (3)
C29—Sn1—O1—Sn2	93.15 (7)	C17—C16—C21—C20	−1.6 (2)
O2—Sn1—O1—Sn2	−0.50 (5)	C15—C16—C21—C20	−178.71 (16)
C33X—Sn1—O1—Sn2	−91.34 (18)	Sn4—O15—C22—O16	5.59 (19)
O4—Sn1—O1—Sn2	174.98 (6)	Sn4—O15—C22—C23	−173.41 (12)
O2—Sn2—O1—Sn3	−175.80 (9)	O16—C22—C23—C24	−177.31 (16)
C41—Sn2—O1—Sn3	−60.08 (9)	O15—C22—C23—C24	1.7 (2)
C37—Sn2—O1—Sn3	84.73 (9)	O16—C22—C23—C28	1.0 (3)
O11—Sn2—O1—Sn3	−150.90 (12)	O15—C22—C23—C28	179.99 (15)
Sn1—Sn2—O1—Sn3	−176.32 (12)	C28—C23—C24—C25	−1.4 (3)
O2—Sn2—O1—Sn1	0.53 (5)	C22—C23—C24—C25	176.97 (16)
C41—Sn2—O1—Sn1	116.24 (7)	C23—C24—C25—C26	0.3 (3)
C37—Sn2—O1—Sn1	−98.95 (7)	C23—C24—C25—N7	−179.13 (16)
O11—Sn2—O1—Sn1	25.42 (19)	O18—N7—C25—C24	178.88 (18)
C53—Sn4—O2—Sn2	−80.21 (10)	O17—N7—C25—C24	−1.0 (3)
C57—Sn4—O2—Sn2	86.76 (10)	O18—N7—C25—C26	−0.6 (3)
O15—Sn4—O2—Sn2	−177.66 (10)	O17—N7—C25—C26	179.57 (18)
O12—Sn4—O2—Sn2	9.00 (9)	C24—C25—C26—C27	0.8 (3)
C53—Sn4—O2—Sn1	115.23 (8)	N7—C25—C26—C27	−179.73 (18)
C57—Sn4—O2—Sn1	−77.80 (8)	C25—C26—C27—C28	−0.9 (3)
O15—Sn4—O2—Sn1	17.79 (7)	C26—C27—C28—N8	−178.89 (19)
O12—Sn4—O2—Sn1	−155.55 (7)	C26—C27—C28—C23	−0.2 (3)
C41—Sn2—O2—Sn4	101.35 (10)	C24—C23—C28—N8	179.97 (17)
C37—Sn2—O2—Sn4	−65.76 (10)	C22—C23—C28—N8	1.7 (3)
O1—Sn2—O2—Sn4	−166.98 (10)	C24—C23—C28—C27	1.3 (3)
O11—Sn2—O2—Sn4	20.39 (9)	C22—C23—C28—C27	−176.99 (16)
Sn1—Sn2—O2—Sn4	−166.48 (13)	O1—Sn1—C29—C30	155.96 (12)
C41—Sn2—O2—Sn1	−92.16 (8)	C33—Sn1—C29—C30	3.2 (3)
C37—Sn2—O2—Sn1	100.72 (7)	O2—Sn1—C29—C30	−125.67 (13)
O1—Sn2—O2—Sn1	−0.50 (5)	C33X—Sn1—C29—C30	−16.9 (3)
O11—Sn2—O2—Sn1	−173.13 (6)	O4—Sn1—C29—C30	69.62 (13)
O1—Sn1—O2—Sn4	169.06 (8)	Sn2—Sn1—C29—C30	−162.57 (12)
C33—Sn1—O2—Sn4	−91.54 (19)	Sn1—C29—C30—C31	−174.93 (13)
C29—Sn1—O2—Sn4	60.51 (8)	C29—C30—C31—C32	−172.63 (17)
C33X—Sn1—O2—Sn4	−84.64 (18)	O1—Sn1—C33—C34	88.2 (4)
O4—Sn1—O2—Sn4	151.52 (17)	C29—Sn1—C33—C34	−117.9 (4)
Sn2—Sn1—O2—Sn4	168.53 (11)	O2—Sn1—C33—C34	9.3 (4)
O1—Sn1—O2—Sn2	0.53 (5)	C33X—Sn1—C33—C34	−26.2 (14)
C33—Sn1—O2—Sn2	99.93 (19)	O4—Sn1—C33—C34	175.7 (4)
C29—Sn1—O2—Sn2	−108.02 (7)	Sn2—Sn1—C33—C34	47.8 (4)
C33X—Sn1—O2—Sn2	106.83 (18)	Sn1—C33—C34—C35	−159.7 (3)
O4—Sn1—O2—Sn2	−17.0 (2)	C33—C34—C35—C36	74.7 (5)
C49X—Sn3—O3—C1	−71.8 (6)	O1—Sn1—C33X—C34X	−4.3 (4)
O1—Sn3—O3—C1	45.20 (16)	C33—Sn1—C33X—C34X	64.2 (16)
C45—Sn3—O3—C1	158.08 (17)	C29—Sn1—C33X—C34X	168.7 (2)
C49—Sn3—O3—C1	−58.71 (17)	O2—Sn1—C33X—C34X	−81.5 (4)
O7—Sn3—O3—C1	79.8 (3)	O4—Sn1—C33X—C34X	85.9 (4)
O1—Sn1—O4—C1	51.15 (17)	Sn2—Sn1—C33X—C34X	−45.0 (4)
C33—Sn1—O4—C1	−51.3 (3)	Sn1—C33X—C34X—C35X	178.7 (3)
C29—Sn1—O4—C1	161.25 (18)	C33X—C34X—C35X—C36X	173.9 (4)
O2—Sn1—O4—C1	68.2 (3)	O2—Sn2—C37—C38	−91.62 (14)
C33X—Sn1—O4—C1	−55.8 (2)	C41—Sn2—C37—C38	106.11 (16)
Sn2—Sn1—O4—C1	55.21 (18)	O1—Sn2—C37—C38	−13.05 (15)
C49X—Sn3—O7—C8	−63.8 (6)	O11—Sn2—C37—C38	−178.45 (14)
O1—Sn3—O7—C8	−179.24 (12)	Sn1—Sn2—C37—C38	−51.34 (15)
C45—Sn3—O7—C8	68.50 (12)	Sn2—C37—C38—C39	161.63 (13)
C49—Sn3—O7—C8	−76.41 (13)	C37—C38—C39—C40	−74.9 (2)
O3—Sn3—O7—C8	145.6 (2)	O2—Sn2—C41—C42	−149.51 (13)
O2—Sn2—O11—C15	−60.74 (14)	C37—Sn2—C41—C42	11.0 (2)
C41—Sn2—O11—C15	−178.00 (14)	O1—Sn2—C41—C42	132.12 (14)
C37—Sn2—O11—C15	41.63 (14)	O11—Sn2—C41—C42	−65.76 (14)
O1—Sn2—O11—C15	−84.9 (2)	Sn1—Sn2—C41—C42	167.68 (12)
Sn1—Sn2—O11—C15	−66.14 (14)	Sn2—C41—C42—C43	176.64 (14)
O2—Sn4—O12—C15	−42.71 (15)	C41—C42—C43—C44	67.4 (2)
C53—Sn4—O12—C15	62.47 (16)	C49X—Sn3—C45—C46	−4.4 (6)
C57—Sn4—O12—C15	−155.51 (16)	O1—Sn3—C45—C46	160.89 (12)
O15—Sn4—O12—C15	−74.1 (3)	C49—Sn3—C45—C46	−8.4 (2)
O2—Sn4—O15—C22	168.90 (11)	O7—Sn3—C45—C46	−118.09 (13)
C53—Sn4—O15—C22	65.04 (12)	O3—Sn3—C45—C46	73.53 (13)
C57—Sn4—O15—C22	−79.97 (11)	Sn3—C45—C46—C47	174.73 (13)
O12—Sn4—O15—C22	−159.18 (18)	C45—C46—C47—C48	−69.0 (2)
Sn1—O4—C1—O3	−38.2 (3)	C49X—Sn3—C49—C50	−22 (2)
Sn1—O4—C1—C2	141.23 (14)	O1—Sn3—C49—C50	−179.25 (16)
Sn3—O3—C1—O4	−22.1 (3)	C45—Sn3—C49—C50	−9.4 (3)
Sn3—O3—C1—C2	158.50 (12)	O7—Sn3—C49—C50	99.38 (17)
O4—C1—C2—C3	−4.8 (3)	O3—Sn3—C49—C50	−88.50 (17)
O3—C1—C2—C3	174.61 (17)	Sn3—C49—C50—C51	67.5 (2)
O4—C1—C2—C7	177.97 (17)	C49—C50—C51—C52	64.9 (3)
O3—C1—C2—C7	−2.6 (3)	O1—Sn3—C49X—C50X	−161.1 (9)
C7—C2—C3—C4	0.4 (3)	C45—Sn3—C49X—C50X	3.6 (14)
C1—C2—C3—C4	−176.93 (17)	C49—Sn3—C49X—C50X	174 (3)
C2—C3—C4—C5	−0.3 (3)	O7—Sn3—C49X—C50X	112.8 (11)
C2—C3—C4—N1	176.96 (16)	O3—Sn3—C49X—C50X	−73.0 (11)
O5—N1—C4—C3	−169.13 (18)	Sn3—C49X—C50X—C51X	166.1 (12)
O6—N1—C4—C3	10.1 (3)	C49X—C50X—C51X—C52X	−70 (2)
O5—N1—C4—C5	8.2 (3)	O2—Sn4—C53—C54	171.92 (13)
O6—N1—C4—C5	−172.65 (18)	C57—Sn4—C53—C54	11.1 (2)
C3—C4—C5—C6	−0.9 (3)	O15—Sn4—C53—C54	−105.84 (14)
N1—C4—C5—C6	−178.09 (17)	O12—Sn4—C53—C54	82.96 (14)
C4—C5—C6—C7	1.9 (3)	Sn4—C53—C54—C55	−60.8 (2)
C5—C6—C7—N2	177.84 (18)	C53—C54—C55—C56	−174.58 (18)
C5—C6—C7—C2	−1.8 (3)	O2—Sn4—C57—C58	99.39 (14)
C3—C2—C7—N2	−178.98 (17)	C53—Sn4—C57—C58	−100.64 (17)
C1—C2—C7—N2	−1.8 (3)	O15—Sn4—C57—C58	16.56 (15)
C3—C2—C7—C6	0.6 (3)	O12—Sn4—C57—C58	−176.11 (15)
C1—C2—C7—C6	177.74 (17)	Sn4—C57—C58—C59	−178.80 (14)
Sn3—O7—C8—O8	1.2 (2)	C57—C58—C59—C60	−178.49 (18)

### Hydrogen-bond geometry (Å, °)

**Table:**

*D*—H···*A*	*D*—H	H···*A*	*D*···*A*	*D*—H···*A*
N2—H1N2···O9^i^	0.86 (3)	2.32 (3)	3.122 (3)	157 (3)
N2—H2N2···O3	0.84 (3)	2.02 (3)	2.684 (3)	135 (2)
N4—H1N4···O6^ii^	0.84 (3)	2.20 (3)	3.002 (2)	160 (3)
N4—H2N4···O8	0.87 (3)	2.02 (3)	2.675 (2)	131 (3)
N6—H1N6···O12	0.82 (3)	2.07 (3)	2.688 (3)	132 (2)
N6—H2N6···O17^iii^	0.83 (3)	2.36 (3)	3.152 (3)	158 (2)
N6—H2N6···O18^iii^	0.83 (3)	2.49 (2)	3.229 (3)	149 (2)
N8—H1N8···O16	0.86 (3)	2.03 (3)	2.674 (2)	131 (3)
N8—H2N8···O13^iv^	0.82 (3)	2.22 (3)	2.998 (2)	160 (3)
C6—H6A···O9^i^	0.95	2.48	3.261 (2)	139
C13—H13A···O6^ii^	0.95	2.57	3.350 (2)	140
C20—H20A···O17^iii^	0.95	2.54	3.338 (3)	142
C43—H43B···O13^v^	0.99	2.58	3.544 (3)	164
C50—H50B···O8	0.99	2.56	3.304 (3)	131
C54—H54A···O16	0.99	2.51	3.210 (3)	128
C58—H58A···O16	0.99	2.58	3.221 (2)	123

Symmetry codes: (i) *x*−1, *y*, *z*; (ii) *x*+1, *y*−1, *z*; (iii) *x*+1, *y*, *z*; (iv) *x*−1, *y*+1, *z*; (v) −*x*+2, −*y*, −*z*+1.
